# The Role of Sesbania grandiflora-Derived Biotin and Bambusa arundinacea-Derived Silica Extracts in Promoting Hair, Skin, and Nail Health: A Randomized, Double-Blind, Placebo-Controlled Clinical Study

**DOI:** 10.7759/cureus.89118

**Published:** 2025-07-31

**Authors:** Maheshvari N Patel, Jit Maheshvari, Nayan Patel

**Affiliations:** 1 Clinical Research Operations, NovoBliss Research Private Limited, Ahmedabad, IND; 2 Pharmacology, Swaminarayan University, Ahmedabad, IND; 3 Research and Development, Orgenetics, Inc., Brea, USA

**Keywords:** bambusa arundinacea, biotin supplementation, hair fall, hair growth, sesbania grandiflora, silica, wrinkles

## Abstract

Background

Biotin, a vital cofactor for carboxylase enzymes, plays a key role in metabolic processes, while silica is believed to support collagen synthesis, contributing to improved skin and hair health. This study investigates the dermatological benefits of standardized plant-based supplements formulated with biotin derived from *Sesbania grandiflora* and a biotin-silica blend extracted from *Bambusa arundinacea. *

Methods

A randomized, double-blind, placebo-controlled clinical trial enrolled 105 participants, with 97 completing the 90-day study. Participants received either Treatment A (placebo), Treatment B (botanical extract of standardised for biotin, 1.25 mg orally administered), or Treatment C (botanical extract of standardised for biotin with silica, 1.25 mg biotin with 21.75 mg silica orally administered). Efficacy was evaluated through clinical and instrumental assessments of hair fall and growth rate, skin elasticity, hydration, wrinkle reduction, and nail texture.

Results

In the post-90-day evaluation, hair fall reduced to 20.61 ± 14.39 (p < 0.001) in biotin and 15.71 ± 10.8 (p < 0.001) in biotin with silica. Hair growth rate increased by 0.55 mm/day (p < 0.0001) in biotin and 0.57 mm/day (p < 0.0001) in biotin with silica. Nail roughness reduced to 0.09 ± 0.29 (p < 0.0001) in biotin and 0.06 ± 0.25 (p < 0.0001) in biotin with silica. Skin elasticity increased to 0.15 ± 0.06 (p < 0.0001) in biotin and 0.17 ± 0.12 (p < 0.0001) in biotin with the silica group. No adverse events were reported.

Conclusion

Incorporating Orgen-Bio® and RGen-Si™ into a daily routine may support overall skin, hair, and nail health. The synergistic blend enhances elasticity, reduces wrinkles, boosts hydration, strengthens hair, and improves nail quality-offering effective, long-term cosmetic and structural benefits.

## Introduction

Biotin, a water-soluble B-complex vitamin, is essential for maintaining healthy hair and skin due to its pivotal role in cellular metabolism [1]. As a coenzyme for carboxylase enzymes, biotin supports the synthesis of fatty acids, amino acids, and glucose, which are vital for nourishing hair follicles and promoting keratin production [2]. This mechanism strengthens the hair shaft, reduces brittleness, and minimizes hair loss. In skin care, biotin enhances lipid metabolism, which helps maintain skin hydration, integrity, and barrier function [3]. Deficiencies in biotin have been linked to symptoms such as thinning hair, brittle nails, and dry, scaly skin, underscoring its importance in dermatological health. Supplementation with biotin has gained attention for its potential to restore hair vitality and improve skin texture, making it a popular ingredient in treatments targeting hair thinning, dullness, and skin dryness [4].

Biotin is an essential coenzyme involved in metabolic processes that support skin and hair health. While severe biotin deficiency is rare, marginal deficiency can occur in certain populations, such as pregnant women or individuals on long-term anticonvulsant therapy. This has led to an increased dermatological interest in biotin supplementation for improving hair strength, reducing brittleness, and enhancing skin health [5].

*Sesbania grandiflora*, rich in bioactive compounds, exhibits strong antioxidant and anti-fibrotic properties, supporting skin and hair health. Its high phenolic and flavonoid content helps reduce oxidative stress, a key factor in aging and hair follicle damage. The extract inhibits activated hepatic stellate cell (HSC-T6) proliferation, lowers hydroxyproline levels, and downregulates fibrogenic cytokines, promoting scalp health and hair growth. These properties make *S. grandiflora* a valuable ingredient in dermatological and hair care formulations [6].

Silica is a trace mineral included in formulations for skin and hair health. It is essential for collagen synthesis, providing structural integrity to skin, hair, and nails. By strengthening connective tissue and improving elasticity, silica may reduce hair shedding and promote overall hair health. When combined with biotin, silica is thought to enhance the benefits: biotin boosts keratin production, while silica supports collagen synthesis, potentially improving skin strength and elasticity. However, there is limited scientific evidence assessing the safety and efficacy of these combinations in controlled human studies [7,8].

This study evaluated the safety and efficacy of plant-based formulations of biotin and biotin-silica blend in healthy adults with hair, skin, and nail concerns, including hair loss, thinning hair, and dry skin. The primary objective was to assess improvements in hair growth, thickness, density, and growth rate, while secondary objectives focused on nail health, skin hydration, skin elasticity, and wrinkle reduction. The evaluations aim to validate the therapeutic potential of these formulations, supporting evidence-based applications for improving hair and skin health.

## Materials and methods

Ethical conduct of the study 

The study adhered to ethical principles, including the Declaration of Helsinki, ICH Good Clinical Practice guidelines, Indian Council of Medical Research (ICMR), and Food Safety and Standards Authority of India (FSSAI). The clinical study involved the continuous use of the assigned test product for a duration of 90 days. The study protocol was approved by the ACEAS-Independent Ethics Committee on June 29, 2023 (approval number: NB230019-OI).

The study was registered with Clinical Trials Registry-India (CTRI: CTRI/2023/11/055359) and ClinicalTrials.gov (NCT05972512) for transparency and public access.

This clinical study has been reported in accordance with the Consolidated Standards of Reporting Trials (CONSORT) guidelines, ensuring transparency and completeness in the presentation of methodology, statistical analysis, and results.

Participants were enrolled after signing an informed consent form, which detailed the study's objectives, methods, confidentiality safeguards, and voluntary participation. The study adhered to a robust ethical framework to protect participants' rights, safety, and well-being and provided them with compensation for every visit.

Study design 

This was a randomized, double-blind, placebo-controlled, single-center, safety, and efficacy study of the plant-based biotin and biotin combined with the plant-based silica. This safety and efficacy study was conducted at NovoBliss Research Private Limited (CRO) Center in Ahmedabad over a treatment duration of 90 days. A total of 105 subjects were randomly assigned to one of three treatment arms: placebo, standardized biotin (1.25 mg), and biotin with silica (1.25 mg biotin + 10 mg silica) based on hair fall and thin, dry, and brittle hair and dry skin complaints. Subject recruitment began on August 22, 2023 (marked by the first subject's first visit) and concluded on December 14, 2023 (marked by the last subject's last visit). Primary endpoints included hair fall, thickness, density, growth rate, and Physicians Global Assessment (PGA) scores of mild, moderate, and severe related to brittle nails. Secondary endpoints included facial wrinkles, skin texture attributes, hydration, hair strength, elasticity, and barrier function.

Courage+Khazaka Electronic GmbH (Cologne, Germany), ISO 9001, 13485-certified bioinstrumentation, widely used in skin and hair research, including space-based studies, was selected for its precision and validated clinical use. These instruments provide accurate, standardized measurements, ensuring consistent and reliable assessment of skin and hair parameters [9,10].

Extraction procedure of standardized biotin and silica

*S. grandiflora* and *B. arundinacea* are botanical water extracts that undergo a water extraction process carried out under specific low heat and pressure conditions to preserve the integrity of the bioactive components and, more importantly, other botanical photo-nutrients and compounds, which may support synergistic activity. These processes also include filtration and concentration steps under a controlled environment to ensure a consistent and standardized assay of actives. The extracts are standardized, such that Orgen-Bio® is to contain not less than (NLT) 0.5% biotin by weight, which is 1.25 mg of biotin (from *S. grandiflora*) per dose, and RGen-Si™ is to contain NLT 75% silica by weight, which is 21.75 mg of silica (from *B. arundinacea*). Each batch is tested in ISO-certified laboratories to confirm the assay of the active constituents (biotin and silica, respectively) and to ensure safety through assessment of impurities such as heavy metals, microbial contaminants, and pesticide residues (Tables 1-2).

**Table 1:** Details about the instruments

Instruments	Manufacturing Details	Software Details	Applications in the Study
Cutometer^®^ Dual MAP 580	Courage+Khazaka Electronic GmbH, Cologne, Germany, 1.16.8	MPA CTplus	To evaluate the skin elasticity and firmness.
Visioscan^®^ VC20 plus		Visioscan	Evaluate four clinical parameters to quantitatively and qualitatively describe the skin surface as an index: Skin smoothness (Sesm), Skin roughness (Ser), Scaliness (Sesc), and Wrinkles (Sew). Skin surface topography provides 3D images
Tewameter^®^ TM Hex		MPA CTplus	To evaluate skin transepidermal water loss to see skin barrier function
CASLite Nova	Catseye Technology, Mumbai, India, DinoCapture 2.0	CASALite Nova	To evaluate the hair growth rate, density and thickness and scalp condition
MoistureMeterEPiD	Delfin Technologies, Kuopio, Finland, 1.1.1.1594	DMC software	To evaluate the accurate moisture level of the skin

**Table 2:** Details about the test treatments

Parameter	Test Treatments	
Test Treatments	Test Treatment A (Placebo): Each blue color capsule contains 300 mg of granulated maltodextrin from tapioca starch (*Manihot esculenta*). Test Treatment B: Each blue color capsule contains 250 mg of *Sesbania grandiflora *extract equivalent to 1.25 mg of biotin. Test Treatment C (the blend of biotin and silica): Each blue color capsule contains 300 mg of a proprietary blend of 250 mg of *Sesbania grandiflora *extract equivalent to 1.25 mg of biotin and 29 mg of *Bambusa arundiancea* extract equivalent to 21.75 mg of silica (10 mg of Si), two times a day after a meal.	
Storage Condition	The test treatments were stored at the study site at room temperature, at 15°C-30°C.	
Dosage Form	Oral blue color capsule.	
Mode of Usage	Two times a day, after each meal	
Route of Administration	The treatment was meant to be administered orally and swallowed as a whole without chewing, with 240 mL of water.	
Manufacturer Details		
Active Ingredients	Orgen-Bio® (*Sesbania grandiflora *extract standardized for 0.5% biotin).	Orgenetics, Inc.
	Orgen-Bio® (*Sesbania grandiflora *extract standardized for 0.5% biotin) and RGen-Si™ (*Bambusa arundinacea *extract standardized for 75% silica).	

Eligibility criteria

The study included healthy subjects, both males and non-pregnant/non-lactating females, aged between 20 and 60 years (inclusive) at the time of consent. Participants presented with complaints of hair fall and thin, dry, and brittle hair, along with dry skin and self-reported non-pathological conditions related to hair. Additionally, individuals who had previously used products for hair thinning were included, provided they agreed to discontinue the use of medicated or prescription shampoos and hair care products containing specific agents such as minoxidil or other anti-thinning compounds. All participants were instructed to exclusively use the designated test treatments throughout the study duration.

The study excluded subjects who had a history of hair thinning due to clinically significant self-reported problems such as anaemia or thyroid issues. Additional exclusion criteria encompassed allergy or sensitivity to test treatment ingredients, any scalp dermatological condition other than hair loss, dandruff, and/or any active dermatological condition that might interfere with the clinical assessments (e.g. tattoos, eczema, psoriasis, acne, etc.), systemic therapy with antibiotics, retinoids, or oral steroids in the prior four weeks, and topical retinoid use within two weeks of the screening.

Randomization and blinding

This study employed a 1:1:1 randomization ratio to allocate participants into three treatment groups: placebo, biotin, and biotin with silica. The randomization sequence was generated using R software (version 4.3.1, 64-bit; R Development Core Team, Vienna, Austria) by an independent biostatistician to ensure unbiased group allocation. Double blinding was maintained by ensuring the study staff who were involved in product dispensing and distribution were not involved in any other study-related activities, and patients were unaware of the test products given to them.

All statistical analyses were performed using R software. The blockrand package (version 1.3) was used for randomization in block random clinical trials (at https://CRAN.R-project.org/package=blockrand).

Study population

A study enrolled 105 healthy male and female subjects aged 20-60 years with hair fall; thin, dry, and brittle hair; dry skin; and self-reported nonpathological conditions related to hair. Exclusion criteria included history of hair thinning, allergies, active dermatological conditions, and chronic illnesses.

Study procedures and visits 

The 90-day study involved eight visits to assess skin, hair, and nail health. The screening visit occurred 30 days before enrollment on day one, where baseline assessments and blood samples were collected. Follow-up visits on days 30, 60, and 90 monitored skin firmness, elasticity, hydration, trans-epidermal water loss (TEWL), hair fall, thickness, volume, and nail health. Hair growth rate evaluations were conducted three days prior to each follow-up visit. The final visit on day 90 completed all assessments and blood tests [11].

Evaluation methods

Standardization for Hair Assessment - Tattoo Method 

Hair assessments were consistently performed on a single, predefined area of the scalp to ensure accuracy and reproducibility of data. A 1 cm² region was marked using medical-grade ink to create a temporary tattoo, serving as a fixed reference point throughout the study duration. Prior to the baseline assessment, the area was shaved to facilitate accurate evaluation. All subsequent measurements of hair growth parameters were conducted at this same site using a phototrichogram, enabling consistent and reliable tracking of changes over the course of the study [12].

Hair Pull Test - Hair Strength

To semi-quantitatively evaluate the dynamics of hair shedding, a standardized hair pull test was conducted during each study visit. Approximately 20-60 hairs were gently grasped near the scalp and pulled along the hair shaft. If more than 10% of the hair is shed, it indicates active telogen hair loss. Results were classified as negative (0-3 hairs), slightly positive (3-6 hairs), or clearly positive (>6 hairs), providing a consistent measure of hair fall progression throughout the study [12]. 

60-Second Hair Combing Test - Hair Fall

The sixty-second hair count method was employed to assess hair shedding over a 60-second combing period. Participants were instructed to flip their hair forward and comb from the back to the front of the scalp over a contrasting-colored sheet for 60 seconds, using their designated study comb. After combing, trained staff collected and counted shed hairs from the comb and sheet, separating hairs with and without bulbs into pre-labelled zip-lock bags. The total hair count was recorded.

CASLite Nova - Hair Density and Thickness

Hair density and thickness were assessed using the tattoo method from a standardized target area located 30 cm posterior to the nose tip at the vertex region of the scalp. A small, semi-permanent tattoo was applied to ensure consistent imaging across time points [13].

Hair thickness and density were measured using the CASLite Nova instrument (Catseye Systems & Solutions Pvt. Ltd; Mumbai, India). For thickness assessment, images were captured at 200× magnification under the ‘Thickness’ tab of the Phototrichogram menu, ensuring visibility of follicles and scalp. A minimum of three to four hair strands were selected using the drag-and-mark technique to calculate average thickness.

For density assessment, images were captured at 60× magnification under the ‘Density’ tab. Terminal (TH) and vellus hairs (Vh) were identified and marked based on the number of hairs per follicular unit. Hair density (hairs/cm²) was computed from the marked area. All images and results were saved for analysis.

Hair Pluck Test - A:T Ratio

Approximately 30 hairs were plucked from the scalp using forceps with protected jaws while maintaining scalp tension to minimize breakage. After sampling, the area was massaged to relieve discomfort. The plucked hairs were aligned on a glass slide and secured with transparent tape. Hair bulbs were examined under 40× magnification, and the number of anagen and telogen hairs was recorded to calculate the A:T ratio [14].

Griffiths Scale - Skin Appearance

The physician’s global assessment (PGA) was conducted by a dermatologist or dermatologist-trained evaluator using the Griffiths Scale (0-8) to evaluate overall facial skin appearance. The scoring was based on visible signs, including dryness, redness, coarse and fine wrinkles, sallowness, roughness, and skin laxity on a severity scale ranging from 0 to 9 [15].

Physician Global Assessment - Brittle Nails 

The PGA was conducted by a dermatologist or dermatologist-trained evaluator using the 0-5 scale. Onychoschizia, characterized by lamellar splitting of the distal nail plate, was assessed for severity based on visible peeling, fragility, and surface flaking [16].

Visioscan VC 20 Plus

Skin surface characteristics were evaluated using the Visioscan® VC 20plus, a high-resolution UVA-light video camera. The SELS® parameters - skin smoothness (Sesm), skin roughness (Ser), scaliness (Sesc), and wrinkles (Sew) - were assessed to quantitatively and qualitatively describe the skin surface. The device also provided skin topography and 3D imaging for detailed surface evaluation.

Cutometer Dual MP 580

Skin elasticity and firmness were assessed using the Cutometer® Dual MPA 580, a globally recognized device for evaluating the mechanical properties of the skin. The instrument measured the skin’s ability to deform and return to its original state after the application of negative pressure. This method provided quantitative data on parameters such as skin firmness, elasticity, and viscoelasticity.

MoistureMeterEpiD

Skin hydration was assessed by measuring changes in water content at the epidermal level to evaluate the moisture-retaining capacity of the skin. This provided valuable information regarding overall skin health and the efficacy of the test product in maintaining or improving hydration. Accurate moisture levels were determined using a non-invasive method, offering insights into the product’s impact on skin barrier function and hydration status. 

Tewameter® TM Hex

Skin barrier function was evaluated by measuring transepidermal water loss (TEWL) using Tewameter®, a widely recognized instrument for TEWL analysis. This non-invasive method quantified the rate of water evaporation from the skin surface, expressed in g/h/m², providing insights into the integrity and functionality of the skin’s barrier.

Statistical analysis

All data were reviewed before analysis to ensure accuracy and completeness. Subjects who were withdrawn from the study were excluded from the statistical analysis. Continuous variables included hair density, hair growth rate, hair thickness, hydration, total hair count, anagen-to-telogen (A:T) ratio, Q parameters, and lamellar onychoschizia. These variables were reported using the number of observations (N), mean, standard deviation (SD), median, minimum, and maximum values. Categorical variables included the PGA score, dermatological assessments, and responses to questionnaires; these were presented as frequencies and percentages, with graphical representations provided where appropriate. Paired t-tests or Wilcoxon signed-rank tests were used to compare baseline and post-treatment means. Between-group comparisons employed independent t-tests or Mann-Whitney U tests, depending on the data.

Statistical analysis was conducted using Statistical Product and Service Solutions (SPSS, version 29.0.1.0; IBM SPSS Statistics for Windows, Armonk, NY) and Microsoft Excel 2019 (Microsoft® Corp., Redmond, WA), at 5% level of significance.

Sample size calculation 

A sample size of 32 subjects per arm was calculated to detect a statistically significant difference in the primary efficacy endpoints with 80% power at a 5% significance level. Consequently, a total of 105 subjects were enrolled in the study to ensure that at least 97 participants completed the trial. This approach provided adequate power to robustly evaluate both primary and secondary outcomes, ensuring the reliability of the study findings.

## Results

Demographics and other baseline characteristics

**Figure 1:**
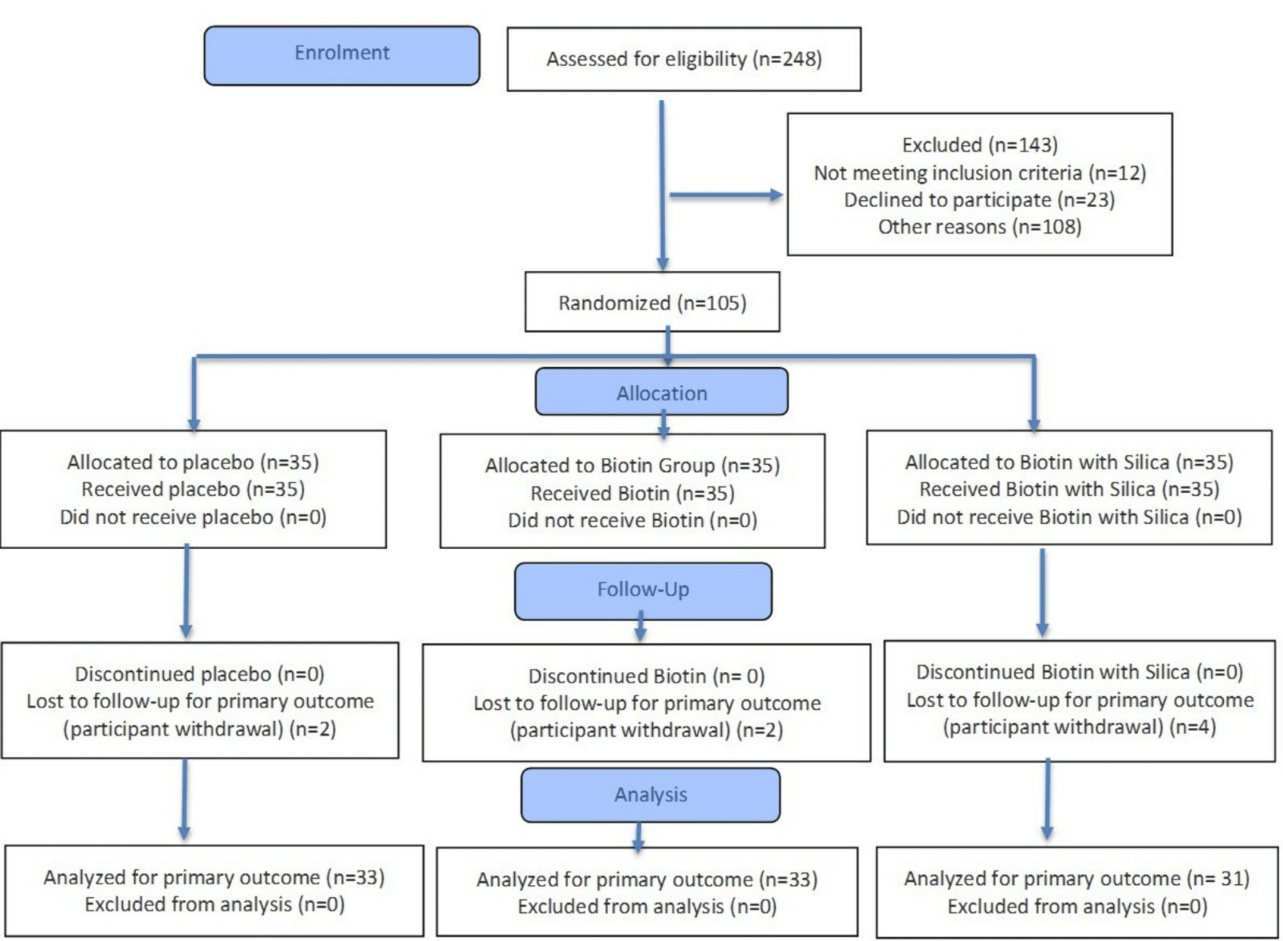
Subject disposition flowchart

The study comprised a total of 105 participants, including 53 females and 52 males. The mean age of the subjects was 40.12 years. Table 3 provides a comprehensive breakdown of demographic characteristics, such as gender, age, weight, and height, distributed across different treatment groups (Figure 1).

**Table 3:** Subject demographics and baseline characteristics SD = Standard deviation

Parameter	Statistic	Placebo	Biotin	Biotin with Silica	Overall
		(N=33)	(N=33)	(N=31)	(N=97)
Gender n (%)	Female	13 (39.39%)	21 (63.64%)	16 (51.61%)	50 (51.55%)
	Male	20 (60.61%)	12 (36.36%)	15 (48.39%)	47 (48.45%)
Race n (%)	Asian	33 (100%)	33 (100%)	31 (100%)	97 (100%)
ConMed History n (%)	No	33 (100%)	33 (100%)	31 (100%)	97 (100%)
Age (Year)	Mean (SD)	37.73 (8.24)	42.03 (8.42)	40.32 (9.05)	40.02 (8.66)
	Median	38	42	39	39
	(Min)	20	29	23	20
	(Max)	58	57	57	58
Weight (kg)	Mean (SD)	64.75 (12)	64.54 (11.58)	63.26 (13.39)	64.20 (2.21)
	Median	65	65	59	64
	(Min)	(36.40)	41	42	(36.40)
	(Max)	101	83	98	101
Height (cm)	Mean (SD)	160.20 (7.25)	156.88 (8.66)	159.16 (9.64)	158.73 (8.57)
	Median	160.2	156	159	158
	(Min)	142	140	143	140
	(Max)	179	174	174	179

Eight subjects were withdrawn from the study due to being lost to follow-up during the further subsequent evaluation visits. Consequently, these subjects were not included in the statistical analysis to maintain the integrity of the per-protocol analysis (Table 3).

Primary efficacy endpoints

Evaluation of Hair Parameters

Assessment of hair fall using the 60-second hair combing method: The assessment of hair fall through the 60-second hair combing method indicated clinically significant differences among the treatment groups. Test Treatment B demonstrated a clinical and highly statistically significant reduction in hair fall of 26.25 ± 19.63 or 23.29% at day 30 (p < 0.001) when compared to the baseline and 20.61 ± 14.39 or 36.19% at day 90 (p < 0.001). Test Treatment C achieved a highly clinical and statistically significant reduction in hair fall of 20.74 ± 13.35 or 23.82% at day 30 (p < 0.001) when compared to the baseline and 15.71 ± 10.8 or 38.89% at day 90 (p = 0.001).

When comparing Test Treatment B with the placebo, a reduction of 162.24% (p < 0.001) was observed, while Test Treatment C showed a reduction of 138.74% (p < 0.001) compared to the placebo at day 90.

Assessment using CASLite Nova through phototrichogram: Changes in hair thickness, as measured using the CASLite Nova through phototrichogram, showed that the placebo group had a non-statistically significant reduction. Test Treatment B demonstrated statistically significant and clinical increases of 18.34 ± 3.09 or 35.5% (p < 0.05) at day 30 when compared to the baseline and 22.61 ± 3.15 or 64.95% (p < 0.05). Test Treatment C demonstrated highly significant increases of 17.48 ± 1.79 or 34.85% (p < 0.0001) at day 30 when compared to the baseline and 22.42 ± 9.1 or 73.67% (p < 0.0001) at day 90.

When comparing Test Treatment B with the placebo, improvement of 491.63% (p < 0.0001) was observed, while Test Treatment C showed improvement of 523.26% (p < 0.01) compared to the placebo.

For hair density, no statistically significant changes were observed in the placebo group. However, Test Treatment B showed clinical and significant increases of 247.12 ± 40.69 or 16.19% (p < 0.0001) at day 30 when compared with the baseline, 316.67 ± 92.53 or 48.16% (p < 0.0001) at day 90. Test Treatment C showed clinical and significant increases of 240.97 ± 36.53 or 13.55% (p < 0.0001) at day 30, when compared with the baseline, 309.00 ± 77.83 or 45.95% (p < 0.0001) at day 90.

When comparing Test Treatment B with the placebo, an increase of 10,512.37% (p < 0.0001) was observed, while Test Treatment C showed an increase of 9,801.03% (p < 0.0001) compared to the placebo.

**Figure 2:**
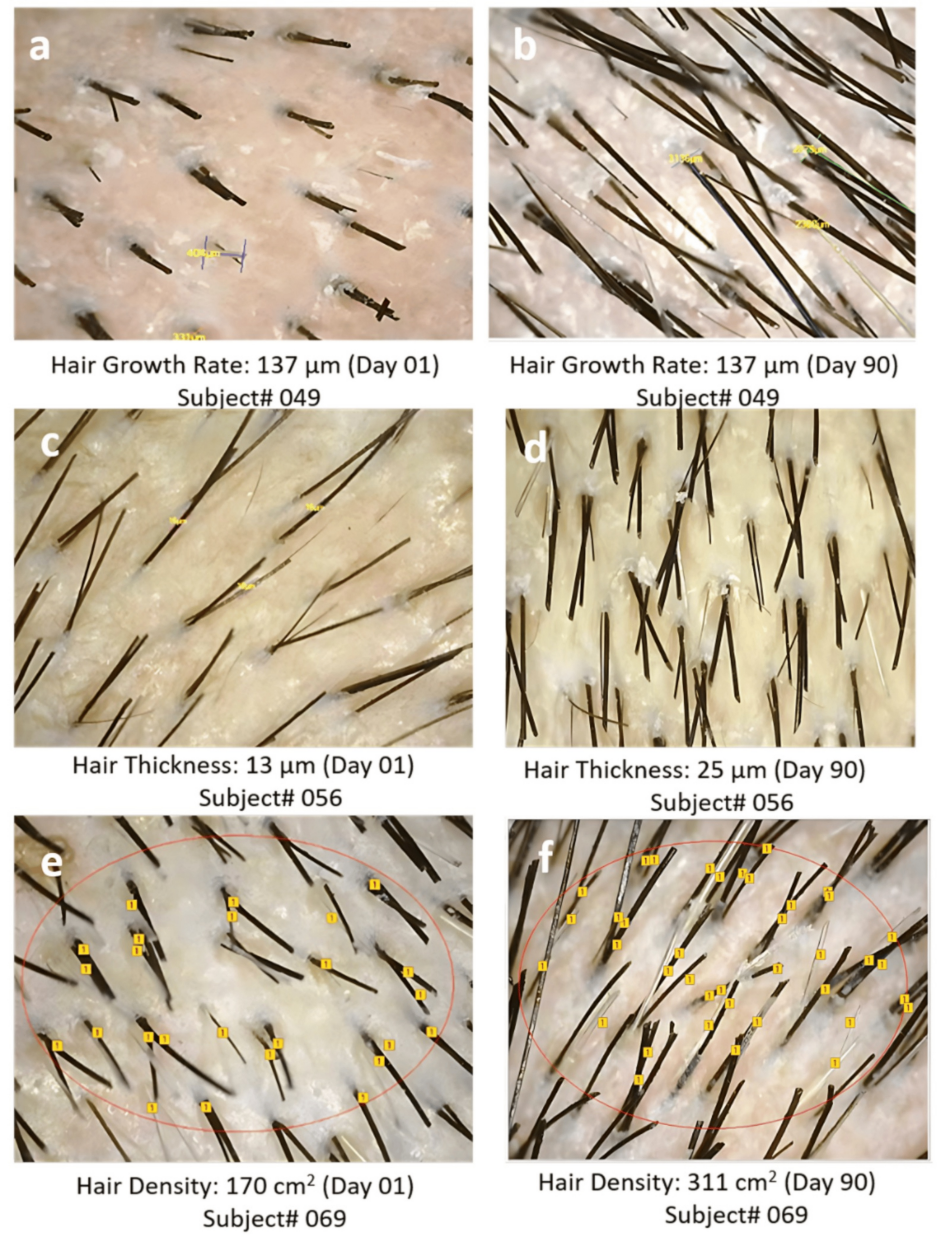
Phototrichograms of the scalp taken using CASLite Nova (a) represents the hair growth rate at day 01, (b) represents the hair growth rate at day 90, (c) represents hair thickness at day 1, (d) represents hair thickness at day 90, (e) represents hair density at day 1, and (f) represents hair density at day 90. In the figure, red markings represent the area selected for the assessment, and yellow ones represent the reading measured by the instrument.

**Figure 3:**
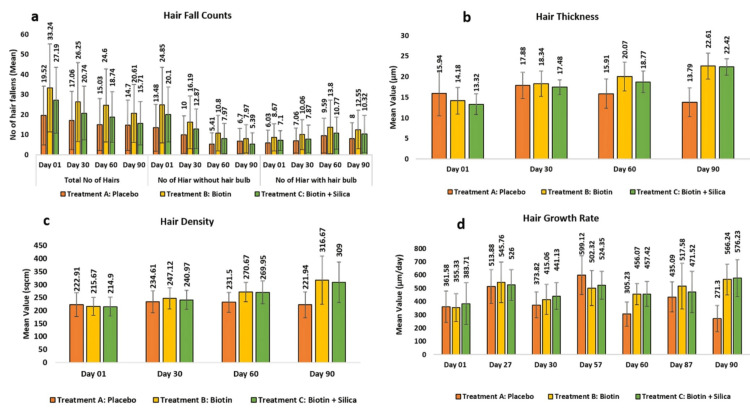
Assessment using CASLite Nova through phototrichogram (a) represents the hair fall counts, (b) represents hair thickness, (c) represents hair density, and (d) represents hair growth rate. The error bars presented in the figures are intended to represent the standard deviation (SD) of the mean values.

The placebo group showed an inconsistent trend in hair growth rate. Test Treatment B consistently increased hair growth rates with a clinical and statistically significant increase of 415.06 ± 113 or 25.99% (p < 0.01) at day 30, when compared to the baseline, 566.24 ± 114.81 or 78.59% (p < 0.0001) at day 90. Test Treatment C consistently increased hair growth rates with a statistically significant increase of 441.13 ± 100.71 or 28.07% (p < 0.01) at day 30, when compared to the baseline, 576.23 ± 137.96 or 73.2% (p < 0.0001) at day 90. The combination of biotin and silica provides synergistic benefits in hair growth more effectively than biotin alone (Figures 2-3). 

Assessment of hair strength using the hair pull test: In the placebo group, the proportion of subjects with good hair strength increased from 21 (63.64%) to 32 (96.97%). For Test Treatments B, subjects with good hair strength increased from 24 (72.73%) to 33 (100.00%), and for Test Treatment C, subjects with good hair strength increased from 13 (41.94%) to 31 (100.00%).

Assessment of anagen and telogen hairs using trichogram: In the placebo group, the A:T ratio was 1.26 ± 1.14 at the baseline, which changed to 1.35 ± 1.45 at day 90, with no statistically significant change (with a confidence interval of -2.57, -0.58). In Test Treatment B, the ratio was 1.65 ± 1.73 at the baseline, which changed to 2.42 ± 1.51 at day 90, with a statistically significant p < 0.05 (with a confidence interval of -0.27, 1.54). In Test Treatment C, the ratio was 1.52 ± 1.62 at the baseline, which changed to 2.34 ± 0.29 at day 90, with a statistically significant p < 0.05 (with a confidence interval of -0.01, -1.88).

**Figure 4:**
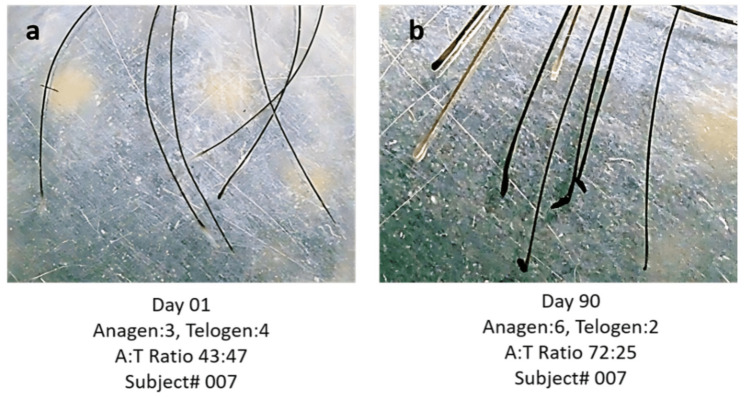
Assessment of the anagen:telogen ratio by a trichogram (a) represents the A:T ratio on day 1 and (b) represents the A:T ratio on day 90.

The A:T ratio is a crucial indicator of hair growth cycle dynamics and directly correlates with the hair growth rate. Hair follicles cycle through distinct phases, anagen (growth phase), catagen (transition phase), and telogen (resting phase) - which regulate hair length, density, and overall scalp coverage. An optimal A:T ratio reflects a healthy hair cycle, where a higher percentage of follicles remain in the anagen phase [17] (Figure 4).

Secondary efficacy endpoints

Evaluation of Nail Parameters

For surface roughness of the nails, the placebo group showed a non-significant reduction. Test Treatment B showed clinical and significant reductions of 0.88 ± 0.49 or 8.62% (p < 0.05) at day 30, when compared to the baseline, 0.09 ± 0.29 or 90% (p < 0.0001) at day 90. Test Treatment C showed reductions of 0.61 ± 0.5 or 13.64% (p < 0.05) at day 30, when compared to the baseline, 0.06 ± 0.25 or 90% (p < 0.0001) at day 90.

When comparing Test Treatment B with the placebo, a reduction of 220.00% (p < 0.0001) was observed, while Test Treatment C showed a reduction of 286.67% (p < 0.0001) compared to the placebo.

For lamellar onychoschizia, the placebo group showed a non-significant reduction. Test Treatment B showed reductions of 1.34 ± 0.7 or 13.44% (p < 0.05) at day 30, when compared to the baseline, and 0.06 ± 0.35 or 96.88% (p < 0.0001) at day 90. Test Treatment C showed reductions of 1.39 ± 0.56 or 10.75% (p < 0.05) at day 30, when compared to the baseline, and 0.13 ± 0.34 or 93.55% (p < 0.0001) at day 90.

**Figure 5:**
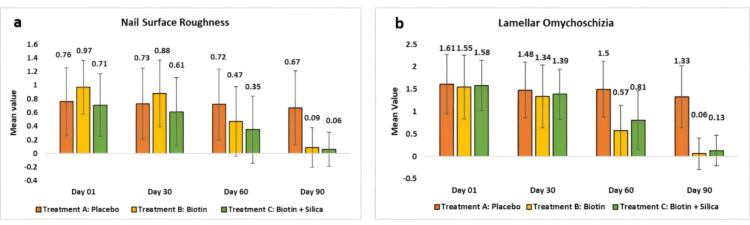
Improvements in the nails observed post 90 days (a) represents surface roughness and (b) represents lamellar onychoschizia.

When comparing Test Treatment B with the placebo, a reduction of 448.15% (p < 0.0001) was observed, while Test Treatment C showed a reduction of 437.04% (p < 0.0001) compared to the placebo at day 90 (Figure 5).

**Figure 6:**
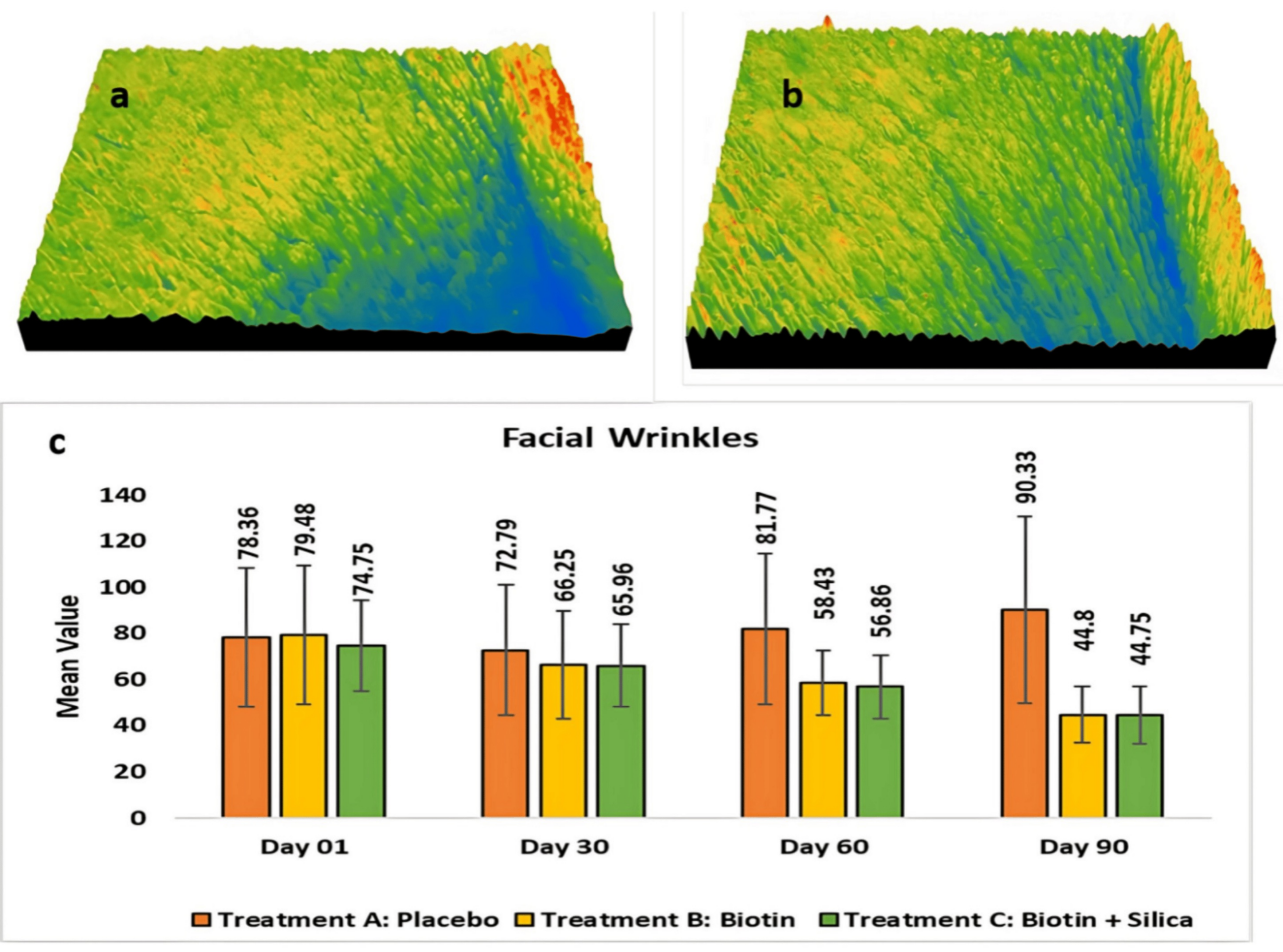
Assessment of crow’s feet area using Visioscan® VC20 Plus (a) represents skin wrinkles at day 01), (b) represents skin wrinkles at day 90), and (c) represents change across all the test treatment from day 01 to day 90.

Assessment of crow’s feet wrinkle reduction using Visioscan® VC20 Plus: The placebo group showed consistent effects on facial wrinkles in the crow's feet area, with an increase of 72.79 ± 28.42 or 6.66% (p < 0.001) at day 30 when compared with the baseline, 90.33 ± 40.35 or 17.25% (p < 0.05) at day 90. In Test Treatment B, there was a clinical and significant reduction of 66.25 ± 23.38 or 14.06% (p < 0.001) at day 30 when compared with the baseline, 44.8 ± 12.17 or 39.97% (p < 0.001) at day 90. In Test Treatment C, there was a reduction of 65.96 ± 17.92 or 11.06% (p < 0.001) at day 30 when compared with the baseline, 44.75 ± 12.4 or 37.76% (p < 0.001) at day 90 (Figure 6).

In the figure above, the blue-highlighted area on day 01 represents wrinkle depth, which was significantly reduced by day 90 following test treatment, as evidenced by the diminished impression of the highlighted area.

Assessment of the skin texture using Visioscan® VC20 Plus: In placebo group, the skin roughness value showed non-significant changes in the placebo group. Test Treatment B showed a significant decrease of 3.02 ± 1.14 or 53.8% (p < 0.05) at day 30 when compared to the baseline, 2.30 ± 1.15 or 20.97% (p < 0.05) at day 90. Test Treatment C showed a significant decrease of 2.7 ± 1.16 or 36.49% (p < 0.05) at day 30 when compared to the baseline, 2.38 ± 1.1 or 27.59% (p < 0.05).

**Figure 7:**
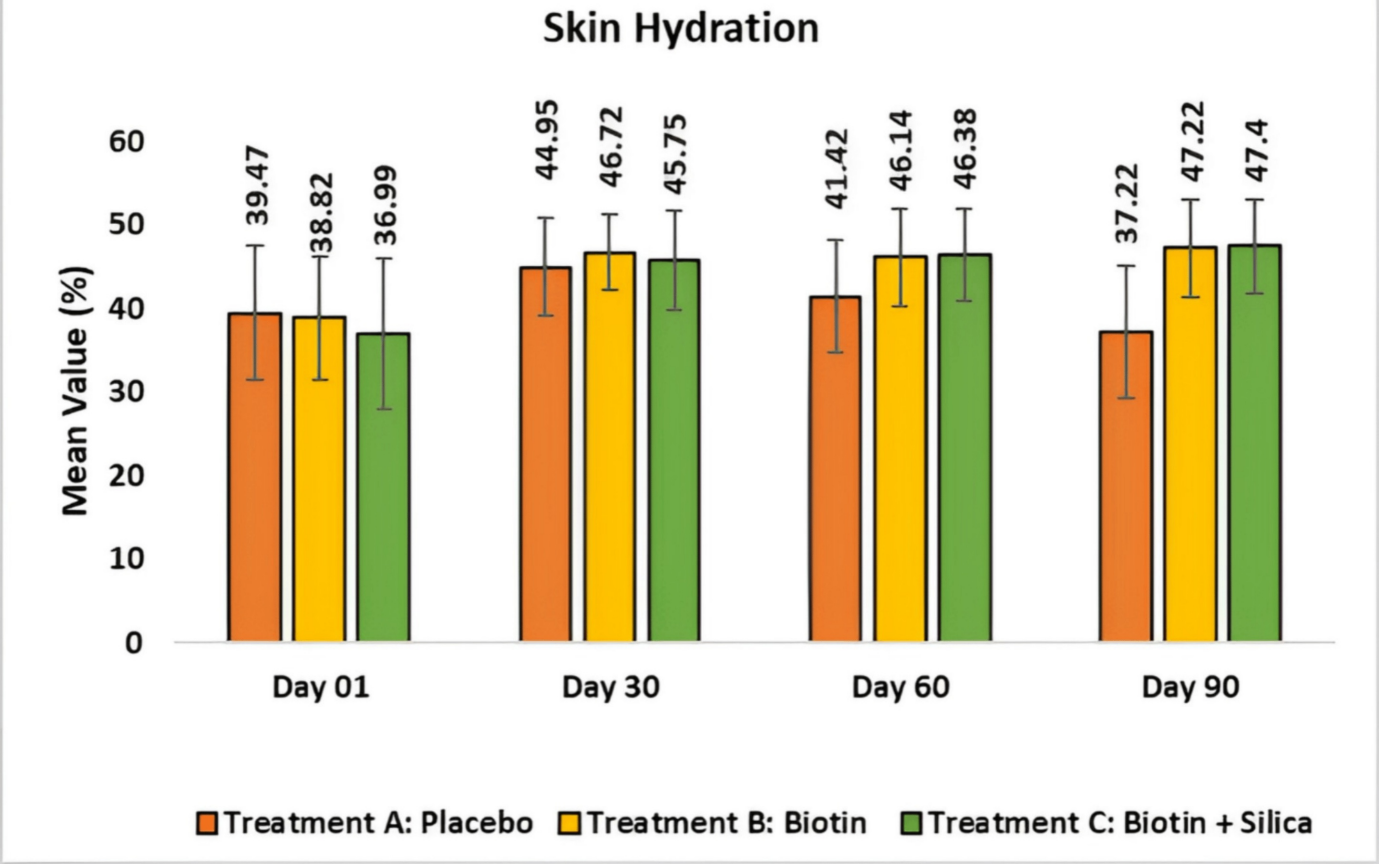
Assessment of skin hydration using MoistureMeterEPiD

Assessment of skin hydration using MoistureMeterEPiD: The placebo group showed a decrease in skin hydration. In Test Treatment B, skin hydration clinically and significantly increased by 46.72 ± 4.54 or 24.55% (p < 0.001) at day 30 when compared to the baseline and 47.22 ± 5.87 or 25.66% (p < 0.0001) at day 90. In Test Treatment C, skin hydration increased by 45.75 ± 5.99 or 29.94% (p < 0.001) at day 30 when compared to the baseline and 47.4 ± 5.51 or 35.42% (p < 0.0001) at day 90. The combination of biotin and silica provides synergistic benefits in enhanced skin hydration than biotin alone (Figure 7).

Assessment of skin elasticity by using Cutometer® Dual MAP 580: The Cutometer® Dual MAP 580 assessment revealed notable changes in skin elasticity parameters (R and Q series) for the placebo and two test treatments (B and C) across multiple visits. The R0 parameter, indicative of skin firmness, showed significant reductions across all groups. In the placebo group, it showed a reduction. In Treatment B, it clinically and statistically reduced to 0.29 ± 0.06 or 23.78 (p < 0.0001) at day 30 when compared to baseline and 0.15 ± 0.06 or 61.86 (p < 0.0001) at day 90. In test treatment C, it reduced to 0.28 ± 0.07 or 23.38 (p < 0.0001) at day 30 when compared to baseline and 0.17 ± 0.12 or 54.01 (p < 0.0001) at day 90.

When comparing Test Treatment B with the placebo, a statistically significant reduction of p < 0.001 was observed, while Test Treatment C showed a reduction of p > 0.05 compared to the placebo.

The Q0 parameter showed significant reductions across all groups. In Treatment B, it clinically and statistically reduced to 0.85 ± 0.18 or 24.42 (p < 0.0001) at day 30 when compared to baseline and 0.43 ± 0.2 or 63.39 (p < 0.0001) at day 90. In test treatment C, it reduced to 0.84 ± 0.2 or 23.46 (p = 0.0001) at day 30 when compared to baseline and 0.45 ± 0.17 or 59.16 (p < 0.0001) at day 90.

**Figure 8:**
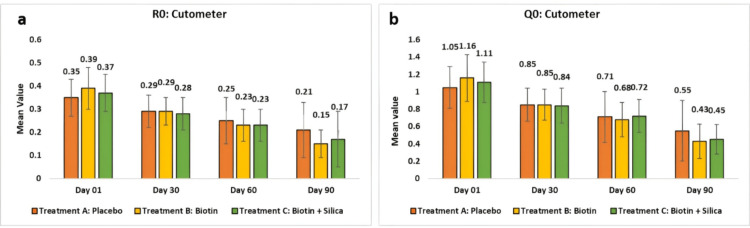
Assessment of skin elasticity using Cutometer® Dual MAP 580 (a) represents the R0 value and (b) represents the Q0 value.

When comparing Test Treatment B with the placebo, a statistically significant reduction of p < 0.01 was observed, while Test Treatment C showed a reduction of p > 0.05 compared to the placebo (Figure 8).

Dermatological assessment using the Griffiths scale: Test treatment B showed a decrease in 1.33 ± 0.25 or 14.41% (p < 0.0001) at day 30 when compared with the baseline, 0.66 ± 0.12 or 56.86% (p < 0.0001) at day 90. Test treatment C showed a decrease in 1.34 ± 0.23 or 13.94% (p < 0.0001) at day 30 when compared with the baseline, 0.66 ± 0.21 or 57.97% (p < 0.0001) at day 90. Both treatments showed effective reductions in dryness, redness, fine lines, coarse wrinkles, laxity, roughness, and sallowness of the skin.

Assessment of skin barrier function using Tewameter® TM Hex: The placebo had a significant decrease of 7.75 ± 2.2 or 16.13% (p < 0.0001) at day 30 when compared with the baseline, 10.49 ± 3.7 or 13.79% (p < 0.05) at day 90. Test Treatment B showed a significant reduction of 7.12 ± 2.41 or 17.95% (p < 0.0001) at day 30 when compared with the baseline, 6.08 ± 1.09 or 22.63% (p < 0.0001) at day 90. Test Treatment C showed a clinically significant reduction of 7.68 ± 2.44 or 16.49% (p < 0.0001) at day 30 when compared with the baseline, 6.29 ± 1.17 or 27.95% (p < 0.0001) at day 90. The combination of biotin and silica provides synergistic benefits in the improvement of skin barrier, indicating a decrease in water loss from the skin surface rather than biotin alone.

Effect of the test treatments in terms of treatment perception: Participants’ perception varied across treatments. By visit eight, Treatment C had the highest likability (72.73%) and satisfaction (58.06%), followed by Treatment B (51.61% likability, 41.94% satisfaction). Treatment A had the lowest scores (28.57% likability, 22.22% satisfaction).

Both Treatments B and C improved skin elasticity and suppleness, with 45.45% and 48.39% of participants rating Treatments C and B as statistically effective by visit eight, respectively. Treatment A showed less improvement (18.75%). Skin suppleness followed a similar trend, with Treatment C at 53.33%, B at 37.93%, and A at 16.67%. Fragrance preference increased for Treatments B (48.15%) and C (60.87%). For moisturization, 63.16% found Treatment C highly moisturizing, followed by 50% for B and 27.78% for A.

Treatments B and C improved skin tone, hair shine, and thickness, with Treatment C leading in skin brightening. Hair shine was highest with Treatment C (57.58%), while Treatment A had the least impact (23.08%). Nail strength and shine also improved more with Treatments B (40%) and C (55.56%) compared to A (22.22%) by visit eight.

The placebo group showed minimal changes in hair volume, shininess, plasticity, reflection, density, and frizziness. In contrast, Test Treatment B demonstrated significant improvements, with 90.91% of subjects showing full hair volume, 87.88% experiencing good shininess, and 96.97% reporting dense hair. Test Treatment C also showed notable benefits, with 87.10% of subjects achieving full hair volume, 93.55% having good shininess, and 93.55% showing dense hair. Both treatments resulted in improved hair quality compared to the placebo.

**Figure 9:**
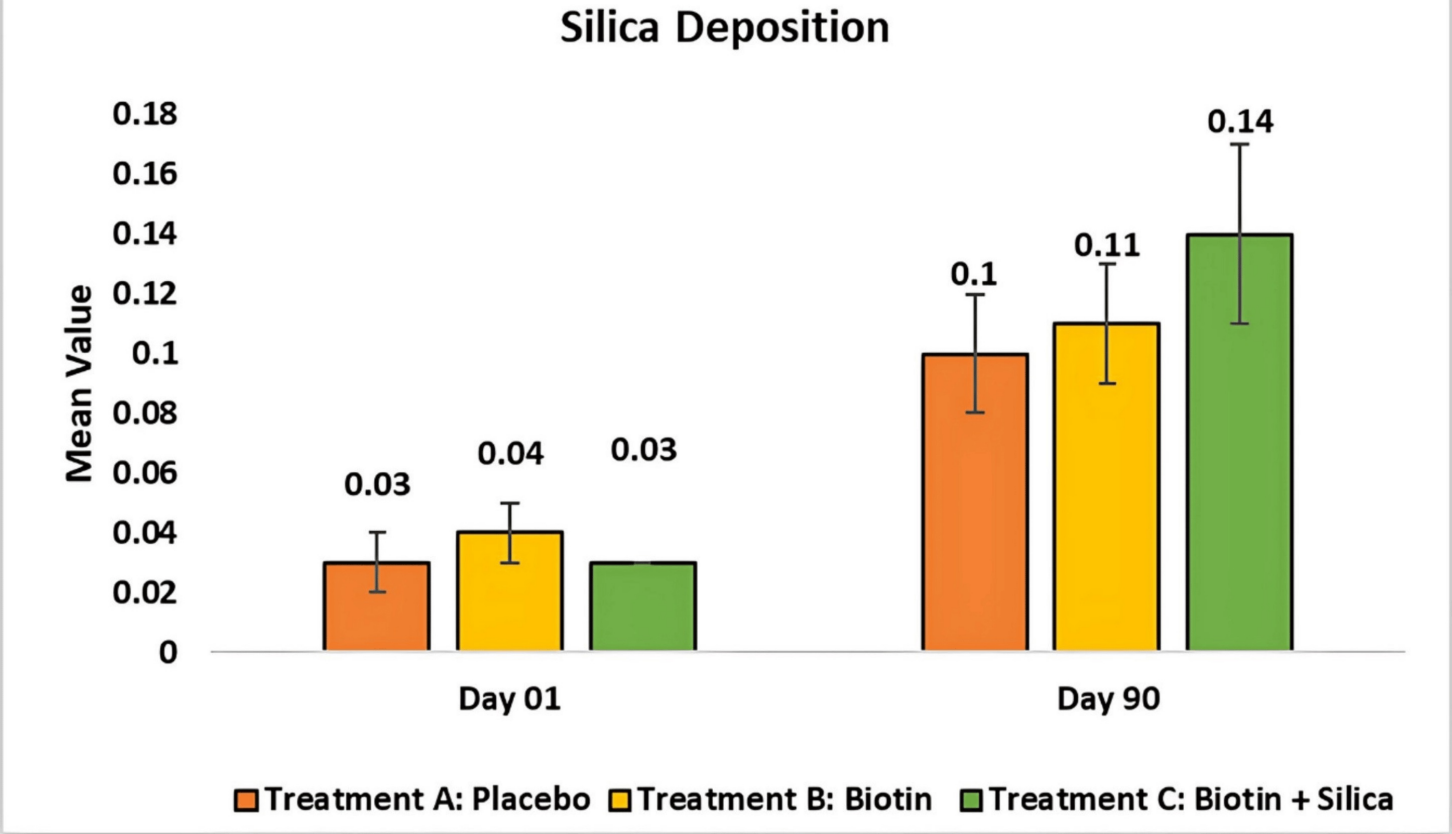
Change in silicon deposits on hair cuticle

Evaluation of silicon deposition on hair cuticle by test treatments using X-ray fluorescence: In this study, the X-ray fluorescence (XRF) technique was used to accurately assess silica deposition in hair, highlighting its role in strengthening hair structure and supporting hair health. The placebo showed an increase in silica of 0.04 ± 0.01 from baseline to 0.1 ± 0.02 at day 90 (p < 0.001), and Test Treatment B showed a statistically significant increase of 0.04 ± 0.01 from baseline to 0.1 ± 0.02 at day 90 (p < 0.001). Test Treatment C showed a statistically significant increase of 0.03 ± 0.00 from baseline to 0.14 ± 0.03 at day 90 (p < 0.0001) (Figure 9).

A comparative analysis was performed to evaluate the safety and efficacy outcomes between Treatment B and Treatment C, highlighting their respective dermatological benefits (Tables 4-5).

**Table 4:** Comparative analysis of biotin and biotin with silica

Parameters	Comparison of Biotin with Biotin + Silica Blend	
	%CFB (Change from Baseline) (Day 90)	P-value (Day 90)
No of Total Hairs	9.18	0.6097
Hair Thickness	8.08	<0.0001
Hair Density	-6.83	>0.05
PGA scores for surface Roughness	26.14	>0.05
Lamellar Onychoschizia	2.03	>0.05
Ridging	-20.83	>0.05

**Table 5:** Descriptive change from baseline to post-baseline in placebo, biotin, and biotin with silica (↑) indicates the increase or enhance in the evaluation parameter, and ↓ indicates the decrease or reduction in the evaluation parameter.

Variables	Visit Days	Placebo			Biotin			Biotin with Silica		
		%CFB	P-Value	Confidence Interval	%CFB	P-Value	Confidence Interval	%CFB	P-Value	Confidence Interval
Hair Parameters										
Hair Fall Counts	Day 30	17.06% ↓	0.1799	-6.1, 1.19	-23.29% ↑	<0.0001	-9.06, -4.82	-23.82% ↑	<0.0001	-8.46, -4.44
	Day 60	15.03% ↓	0.0079	-7.79, -1.28	-30.37% ↑	<0.0001	-11.94, -7	-29.88% ↑	<0.0001	-10.92, -5.99
	Day 90	14.7% ↓	0.0040	-7.99, -1.65	-36.19% ↑	<0.0001	-16.05, -9.23	-38.89% ↑	<0.0001	-14.54, -8.43
Hair Thickness	Day 30	18.41% ↓	0.0553	-0.05, 3.93	35.5% ↑	<0.0001	2.78, 5.78	34.85% ↑	<0.0001	3.31, 5.01
	Day 60	6.76% ↓	1.0000	-2.22, 2.22	47.71% ↑	<0.0001	4.45, 7.55	44.43% ↑	<0.0001	4.44, 6.46
	Day 90	-8.41% ↓	0.0393	-4.19, -0.11	64.95% ↑	<0.0001	7.07, 9.78	73.67% ↑	<0.0001	8.03, 10.16
Hair Density	Day 30	6.87% ↓	0.0703	-1.03, 24.42	16.19% ↑	<0.0001	22.38, 44.12	13.55% ↑	<0.0001	15, 37.13
	Day 60	6.99% ↓	0.3090	-8.75, 26.75	26.31% ↑	<0.0001	39.42, 67.05	27.96% ↑	<0.0001	38.25, 71.85
	Day 90	2.44% ↓	0.9244	-21.62, 19.68	48.16 % ↑	<0.0001	69.69, 132.31	45.95% ↑	<0.0001	66.68, 121.51
Hair Growth Rate	Day 30	10.2% ↓	0.4162	-18.03, 42.51	25.99% ↑	0.0095	15.94, 105.43	28.07% ↑	0.00433	19.42, 95.42
	Day 60	-9.05% ↓	0.0064	-88.6, -15.85	42.14% ↑	0.0001	52.92, 145.21	35.88% ↑	0.0082	20.52, 126.9
	Day 90	-21.56% ↓	<0.0001	-124.85, -55.69	78.59% ↑	<0.0001	153.3, 268.51	73.2% ↑	<0.0001	125.11, 259.92
A:T Ratio	Day 01	-	-	-	-	-	-	-	-	-
	Day 90	36.76% ↑	0.9038	-2.3, -0.97, -2.57, -0.58	143.94% ↑	0.1622	-1.45, 0.11, -3.13, -1.41	138.96% ↑	0.0472	-0.92, 0.54, -3.51, -1.78
Silica Deposition to Hair Cuticle	Day 01	-	-	-		--	-	-	-	-
	Day 90	213.74% ↑	0.0002	0.05, 0.09	194.27% ↑	0.0001	0.05, 0.09	342.68% ↑	<0.0001	0.08, 0.13
Nails Parameters										
Lamellar Onychoschizia	Day 30	-5.56% ↑	0.0719	-0.24, 0.00	-13.44% ↑	0.0107	-0.37, -0.07	-10.75% ↑	0.0197	-0.34, -0.05
	Day 60	-5.73% ↑	0.0719	-0.25, 0.00	-64.37% ↑	<0.0001	-1.20, -0.74	-52.69% ↑	<0.0001	-0.96, 0.59
	Day 90	-13.13% ↑	0.0177	-0.48, -0.07	-96.88 % ↑	<0.0001	-1.75, -1.22	-93.55% ↑	<0.0001	-1.66, -1.24
Roughness	Day 30	-4.17% ↑	1.0000	-0.09, 0	-8.62% ↑	0.2330	-0.29, -0.18	-13.64% ↑	0.1489	-0.31, -0.16
	Day 60	-8.70% ↑	0.7728	-0.11, -0.01	-50% ↑	0.00031	-0.59, -0.45	-50% ↑	0.0011	-0.6, -0.45
	Day 90	-12.5% ↑	0.1489	-0.12, 0.02	-90% ↑	<0.0001	[-2.59, 8.7	-90.91% ↑	<0.0001	-1, -0.82
Ridging	Day 30	-10.53% ↑	0.1489	-0.19, 0.01	-16.67% ↑	0.1736	-0.23, 0.05]	-16.67% ↑	0.1736	-0.29, 0.03
	Day 60	-8.33% ↑	0.3458	-0.15, 0.03	-72.92% ↑	0.0009	-0.52, -0.14	-72.92% ↑	0.0009	-0.63, -0.21
	Day 90	-21.05% ↑	0.0369	-0.28, -0.02	-100.00% ↑	0.0001	-0.66, -0.30	-100.00% ↑	0.0001	-0.83, -0.33
Skin Parameters										
Skin Hydration	Day 30	18.65% ↓	0.0003	2.75, f8.22	24.55% ↑	<0.0001	5.5, 10.74	29.94% ↑	<0.0001	7.2, 13.6
	Day 60	10.28% ↓	0.2782	-1.63, 5.46	21.6% ↑	<0.0001	3.71, 10.19	32.25% ↑	<0.0001	6.29, 12.48
	Day 90	-3.31% ↓	0.1350	-5.25, 0.74	25.66% ↑	<0.0001	5.35, 11.45	35.42% ↑	<0.0001	5.8, 11.72
Crow’s Feet Wrinkles	Day 30	-6.66% ↑	0.00027	-8.34, -2.8	-14.06% ↑	<0.0001	-15.61, -8.18	-11.06 ↑	<0.0001	-12.44, -5.14
	Day 60	5.71% ↓	0.34770	-3.45, 9.51	-23.11% ↓	<0.0001	-29.74, -13.05	-22.24% ↓	<0.0001	-22.78, -13
	Day 90	17.25% ↓	0.03930	0.62, 23.31	-39.97% ↓	<0.0001	-43.64, -25.73	-37.76% ↓	<0.0001	-36.85, -23.15
Skin Elasticity	Day 30	-15.75% ↑	0.0001	-0.09, -0.03	-23.78% ↑	<0.0001	-0.13, -0.07	-23.38% ↑	<0.0001	-0.12, -0.06
	Day 60	-25.67% ↑	0.0001	-0.14, -0.05	-41.76% ↑	<0.0001	-0.2, -0.14	-37.13% ↑	<0.0001	-0.17, -0.11
	Day 90	-37.16% ↑	0.0001	-0.19, -0.09	-61.86% ↑	<0.0001	-0.26, -0.21	-54.01% ↑	<0.0001	-0.25, -0.16
Skin Barrier Function	Day 30	-16.13%	<0.0001	-3.11, -1.25	-17.95%	<0.0001	-2.86, -1.16	-16.49%	0.0001	-3.55, -1.31
	Day 60	-4.59% ↑	0.0256	-2.3, -0.16	-16.75% ↑	0.0002	-3.39, -1.16	-14.61% ↑	0.0005	-4.05, -1.26
	Day 90	13.79% ↑	0.4198	-0.83, 1.93	-22.63% ↑	<0.0001	-3.97, -1.75	-27.9%5 ↑	<0.0001	-5.21, -2.42
Skin Roughness	Day 30	26.19% ↑	0.1811	-0.11, 0.58	53.8% ↑	0.0004	0.39, 1.08	36.49% ↑	0.6537	-0.39, 0.61
	Day 60	8.20% ↓	0.1924	-0.67, 0.14	32.1% ↓	0.2475	-0.14, 0.8	23.28% ↓	0.5219	-0.61, 0.32
	Day 90	-0.24% ↓	0.1531	-0.72, 0.12	20.97% ↓	0.8969	-0.45, 0.51	27.59% ↓	0.4387	-0.75, 0.33

## Discussion

This 90-day clinical study evaluated the effectiveness of three treatments on skin and hair health parameters: placebo (Test Treatment A), biotin (Test Treatment B), and a combination of biotin with silica (Test Treatment C).

Biotin and silica are widely recognized for their benefits in addressing hair loss by stimulating keratin production, promoting hair growth, and preventing hair thinning, with potential effects in reversing hair loss. Silica, in particular, strengthens hair by delivering essential nutrients to hair follicles. Silica is thought to strengthen hair and prevent thinning by supplying essential nutrients to hair follicles [18]. The average human hair growth rate is approximately 350 µm/day [19,20].

Biotin and silica work synergistically to promote healthy hair and skin through complementary biological mechanisms. Biotin, a water-soluble B vitamin, plays a crucial role in keratin production, the primary structural protein found in hair, skin, and nails. It acts as a coenzyme in the metabolism of fatty acids and amino acids, which are vital for maintaining the structural integrity and strength of these tissues. On the other hand, silica, a trace mineral, supports the synthesis of collagen, a key component of the skin’s extracellular matrix that provides firmness and elasticity. Silica also enhances the delivery of essential nutrients to the hair follicles by improving blood circulation and strengthening connective tissues. When combined, biotin and silica amplify each other’s effects - biotin promotes the growth and maintenance of keratin-rich tissues, while silica supports the underlying collagen framework -together fostering improved hair thickness, resilience, and skin texture [21].

This study is novel in its evaluation of a standardized blend of plant-based biotin from *S. grandiflora* and silica from *B. arundinacea*, unlike previous studies that primarily assessed synthetic or isolated forms. The use of natural, bioavailable sources, combined with a robust design and both subjective and instrumental evaluations, provides valuable insights into their synergistic effects on hair, skin, and nail health.

Interestingly, a modest improvement in hair thickness, density, and growth rate was observed in the placebo group at day 30 compared to baseline. This may be attributed to psychological or behavioral factors such as increased self-care, dietary awareness, or a placebo effect triggered by participants’ expectations. Seasonal variations and the natural hair growth cycle could also have contributed to these transient changes.

The study demonstrated the efficacy of Test Treatments B and C in significantly improving parameters related to hair fall, hair growth, skin, and nail health compared to the placebo group. The results highlight the superiority of the test treatments in addressing hair and skin health concerns, with Test Treatment C often showing slightly better outcomes than Test Treatment B across several parameters. The findings align with previous research that suggests biotin's positive effects on skin health, reducing dryness and enhancing hydration [22].

Test Treatments B and C were statistically and clinically effective in reducing hair fall, as measured using the 60-second hair combing method. Both treatments demonstrated consistent improvements over time. Test Treatment B resulted in a notable reduction in total hair fall, while Test Treatment C achieved a comparable reduction. The reductions in hair fall for hairs with bulbs and without bulbs also indicated a greater efficacy for the test treatments compared to the placebo. These findings suggest that the active ingredients in Test Treatments B and C may enhance hair follicle stability and reduce shedding. Moreover, phototrichogram analysis revealed a significant increase in hair density and thickness with both test treatments. Test Treatment B achieved a substantial increase in hair thickness and density, while Test Treatment C showed even greater improvements. These results indicate that both treatments may promote the regeneration of hair follicles and improve the overall quality of hair.

In addition to hair benefits, both Test Treatments B and C significantly improved nail parameters, particularly surface roughness and lamellar onychoschizia. Test Treatment B markedly reduced nail surface roughness, while Test Treatment C achieved a comparable reduction. For lamellar onychoschizia, both test treatments showed improvements, indicating enhanced nail strength and integrity. Skin texture and hydration also showed substantial improvements with both treatments. Skin texture assessment demonstrated that both test treatments effectively reduced skin roughness, with improvements sustained over time. Skin hydration analysis showed significant increases with both test treatments, with Test Treatment C consistently performing better. This indicates that the active components in both treatments are effective in enhancing the skin's moisture-retention capacity, likely due to improved skin barrier function.

The analysis of facial wrinkles in the crow's feet area further supported the effectiveness of the test treatments. Both Test Treatments B and C significantly reduced wrinkle severity over time. These results highlight the potential anti-aging properties of the test treatments. While Test Treatment C often showed marginally better performance, both treatments were consistent in their efficacy, underscoring the potency of their active ingredients. This effect can be attributed to the synergistic roles of biotin, a key B vitamin for skin health, and silica, which supports collagen formation and skin hydration.

The observed reduction in low-density lipoprotein (LDL) cholesterol and total cholesterol with Test Treatments B and C aligns with existing literature on biotin's impact on lipid metabolism, suggesting potential systemic benefits beyond dermatological outcomes [23]. Importantly, no adverse effects were observed in blood parameters, supporting the safety of these interventions. The placebo group showed a significant but comparatively lower improvement in silica deposition than the treatment group, emphasizing the enhanced efficacy of the active intervention.

Several studies have explored the efficacy of biotin and silicon supplementation in enhancing hair and skin health. An open-label experience trial evaluated the effects of a novel biotin and silicon supplement on hair and skin. Preclinical studies indicated improvements in the hair growth cycle, increased collagen levels in the skin, and enhanced skin elasticity and texture. In a three-month, double-blind, placebo-controlled trial involving 90 women, the supplement increased hair thickness and reduced wrinkles compared to the placebo [24].

Another clinical trial is currently underway to evaluate the safety and effectiveness of plant-based biotin and plant-based biotin with silica in healthy adults experiencing hair fall, thin, dry, and brittle hair, and dry skin [21]. This randomized, double-blind, placebo-controlled study aims to provide further insights into the potential benefits of these supplements.

The study demonstrates the potential of the test treatments to provide notable improvements in the assessed parameters, highlighting their efficacy and safety. The outcomes align with previous evidence supporting the benefits of the active ingredients, reinforcing their role in promoting health and well-being. Despite certain limitations, such as the study's sample size and duration, the results provide a strong foundation for future research related to the evaluation in the targeted diverse population. These findings underscore the promise of the test treatments, encouraging further exploration in larger, long-term studies to confirm their sustained benefits and mechanisms of action.

## Conclusions

The study demonstrated that the biotin-silica combination exhibited superior efficacy compared to biotin alone and placebo in enhancing skin, hair, and nail health. The synergistic interaction between biotin and silica significantly improved skin elasticity and hydration, strengthened hair, and reduced nail roughness and ridging. Additionally, the treatment positively influenced the hair growth cycle by promoting the anagen (growth) phase, leading to improved hair growth and density. These findings highlight the therapeutic potential of this plant-based biotin-silica formulation as a safe and effective intervention for dermatological health. Furthermore, the results support its application in the development of advanced dermatology and cosmetic products targeting comprehensive skin, hair, and nail care.
